# A novel nomogram integrating body composition and inflammatory-nutritional markers for predicting postoperative complications in patients with adhesive small bowel obstruction

**DOI:** 10.3389/fnut.2024.1345570

**Published:** 2024-04-19

**Authors:** Zhibo Wang, Baoying Sun, Yimiao Yu, Jingnong Liu, Duo Li, Yun Lu, Ruiqing Liu

**Affiliations:** ^1^Department of Gastroenterological Surgery, The Affiliated Hospital of Qingdao University, Qingdao, China; ^2^Neurology Department, Central Hospital Affiliated to Shandong First Medical University, Jinan, China; ^3^Department of Radiation Oncology, The Affiliated Hospital of Qingdao University, Qingdao, China; ^4^Institute of Nutrition and Health, College of Public Health, Qingdao University, Qingdao, China

**Keywords:** body composition, inflammatory-nutritional markers, adhesive small bowel obstruction, postoperative complications, prediction

## Abstract

**Background:**

Postoperative complications in adhesive small bowel obstruction (ASBO) significantly escalate healthcare costs and prolong hospital stays. This study endeavors to construct a nomogram that synergizes computed tomography (CT) body composition data with inflammatory-nutritional markers to forecast postoperative complications in ASBO.

**Methods:**

The study’s internal cohort consisted of 190 ASBO patients recruited from October 2017 to November 2021, subsequently partitioned into training (*n* = 133) and internal validation (*n* = 57) groups at a 7:3 ratio. An additional external cohort comprised 52 patients. Body composition assessments were conducted at the third lumbar vertebral level utilizing CT images. Baseline characteristics alongside systemic inflammatory responses were meticulously documented. Through univariable and multivariable regression analyses, risk factors pertinent to postoperative complications were identified, culminating in the creation of a predictive nomogram. The nomogram’s precision was appraised using the concordance index (C-index) and the area under the receiver operating characteristic (ROC) curve.

**Results:**

Postoperative complications were observed in 65 (48.87%), 26 (45.61%), and 22 (42.31%) patients across the three cohorts, respectively. Multivariate analysis revealed that nutrition risk score (NRS), intestinal strangulation, skeletal muscle index (SMI), subcutaneous fat index (SFI), neutrophil-lymphocyte ratio (NLR), and lymphocyte-monocyte ratio (LMR) were independently predictive of postoperative complications. These preoperative indicators were integral to the nomogram’s formulation. The model, amalgamating body composition and inflammatory-nutritional indices, demonstrated superior performance: the internal training set exhibited a 0.878 AUC (95% CI, 0.802–0.954), 0.755 accuracy, and 0.625 sensitivity; the internal validation set displayed a 0.831 AUC (95% CI, 0.675–0.986), 0.818 accuracy, and 0.812 sensitivity. In the external cohort, the model yielded an AUC of 0.886 (95% CI, 0.799–0.974), 0.808 accuracy, and 0.909 sensitivity. Calibration curves affirmed a strong concordance between predicted outcomes and actual events. Decision curve analysis substantiated that the model could confer benefits on patients with ASBO.

**Conclusion:**

A rigorously developed and validated nomogram that incorporates body composition and inflammatory-nutritional indices proves to be a valuable tool for anticipating postoperative complications in ASBO patients, thus facilitating enhanced clinical decision-making.

## Introduction

Adhesive small bowel obstruction (ASBO) ranks as a leading cause of emergency hospital admissions and surgeries (1). Despite advancements in surgical methods, treatment for ASBO patients may lead to extended hospital stays, increased healthcare costs, and notably high morbidity (48%) and mortality rates (5%) (2, 3). The incidence of postoperative complications significantly impacts patients’ postoperative quality of life, an essential metric in evaluating therapeutic effectiveness. Clavien et al. introduced a surgical complications classification system that aids in accurately assessing outcomes across different treatment approaches (4). While the American College of Surgeons National Surgical Quality Improvement Project (NSQIP) is a prevalent risk prediction tool, its complexity and potential inaccuracies render it less suitable for specific patient populations (5).

To date, no universally accepted risk prediction system exists for ASBO patients. Developing a model to forecast postoperative complications and identify risk factors is crucial. Growing evidence suggests that the prognosis and progression of bowel obstruction are linked not only to bowel dysfunction but also to systemic inflammatory responses (6–8). The persistent obstruction leads to digestive tract dilation, intestinal barrier compromise, microbial translocation, and infiltration of inflammatory cells like neutrophils, lymphocytes, platelets, and monocytes, indicative of inflammatory responses in clinical settings (9, 10). Recent research has explored the connection between patient outcomes and various inflammatory-nutritional scores in small bowel obstruction cases (11, 12). These studies have examined scores such as the neutrophil–lymphocyte ratio (NLR), platelet–lymphocyte ratio (PLR), monocyte–lymphocyte ratio (MLR), the albumin–alkaline phosphatase ratio (ALP), systemic immune-inflammation index (SII), and prognostic nutritional index (PNI). These calculated indicators, including NLR and PLR, have proven more sensitive than singular hematological markers like C-reactive protein (CRP) or lymphocyte count in reflecting the inflammatory response and predicting disease progression (13).

Nutritional status is a critical determinant for ASBO, influencing disease progression and patient prognosis (14, 15). Lee et al. demonstrated that nutritional data, such as body mass index (BMI) and weight loss, are correlated with an increased risk of major complications in ASBO patients (16). However, this study did not provide detailed quantitative insights into nutritional status. The widespread adoption of computed tomography (CT) has advanced body composition research, offering more granular insights than traditional metrics like BMI and weight fluctuation (17). CT-based multiple body composition parameters are usually obtained from the images at the level of third lumbar vertebra (L3), which focus on skeletal muscle and adipose tissue (18). These parameters offer superior informativeness in defining nutrition related disorders such as sarcopenia, visceral obesity and sarcopenic obesity, and are associated with adverse outcomes in several gastrointestinal diseases (19, 20).

Despite numerous studies exploring the links between individual nutritional and inflammatory markers and surgical outcomes, comprehensive research integrating laboratory and CT-derived body composition data for ASBO patients is scant. We conducted a systematic and thorough collection of body composition and systemic inflammatory markers (NLR, PLR, LMR, SII, PNI) data to explore their associations. Furthermore, a nomogram was developed to ascertain their predictive capacity for postoperative complications in ASBO patients.

## Materials and methods

### Patients

**Figure 1:**
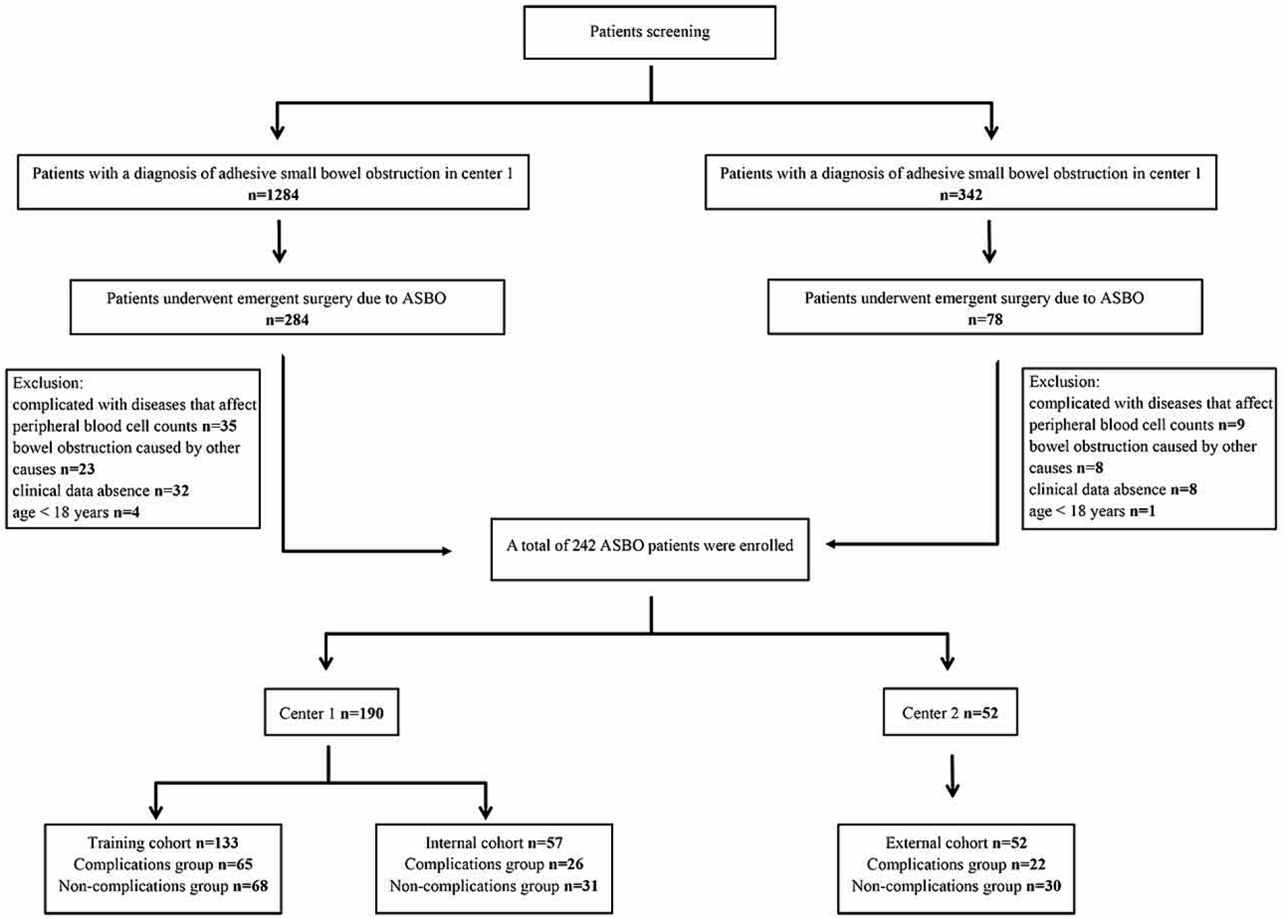
Flowchart for selecting the study population.

This study adheres to the principles of the Declaration of Helsinki. We retrospectively reviewed cases of ASBO from October 2017 to November 2021, utilizing our center’s clinicopathologic database. The inclusion criteria were: (1) diagnosis of ASBO based on clinical or radiological evidence; (2) undergoing emergent surgery due to ASBO; (3) availability of abdominal CT scans and comprehensive hematological indices during hospitalization preoperatively. Exclusion criteria included: (1) conditions that could affect peripheral blood cell counts, such as autoimmune diseases, leukemia, and other hematological malignancies; (2) small bowel obstruction due to primary tumors, hernias, or inflammatory bowel disease; (3) lack of complete clinical data; and (4) age under 18 years. The participants were subsequently divided into training (*n* = 133) and internal validation (*n* = 57) cohorts at a 7:3 ratio. A total of 52 patients were enrolled in the external validation cohort from the Central Hospital Affiliated to Shandong First Medical University between January 2022 and January 2023. Figure 1 illustrates the flowchart of patient selection.

### Date collection

This retrospective study extracted basic clinical data, including age, sex, BMI, symptoms, comorbidities, nutritional risk score (NRS), American Society of Anesthesiologists (ASA) score, intraoperative findings, and related laboratory indicators from the de-identified database and electronic medical record system. Inflammatory-nutritional markers were determined as follows: NLR = N/L, PLR = P/L, LMR = L/M; SII=P × N/L; PNI = albumin (g/L) +5 × L (109/L), where N: neutrophil count, L: lymphocyte [109]/L; P: platelet count, M: monocyte count (21, 22).

### Evaluation of CT-based body composition

**Figure 2:**
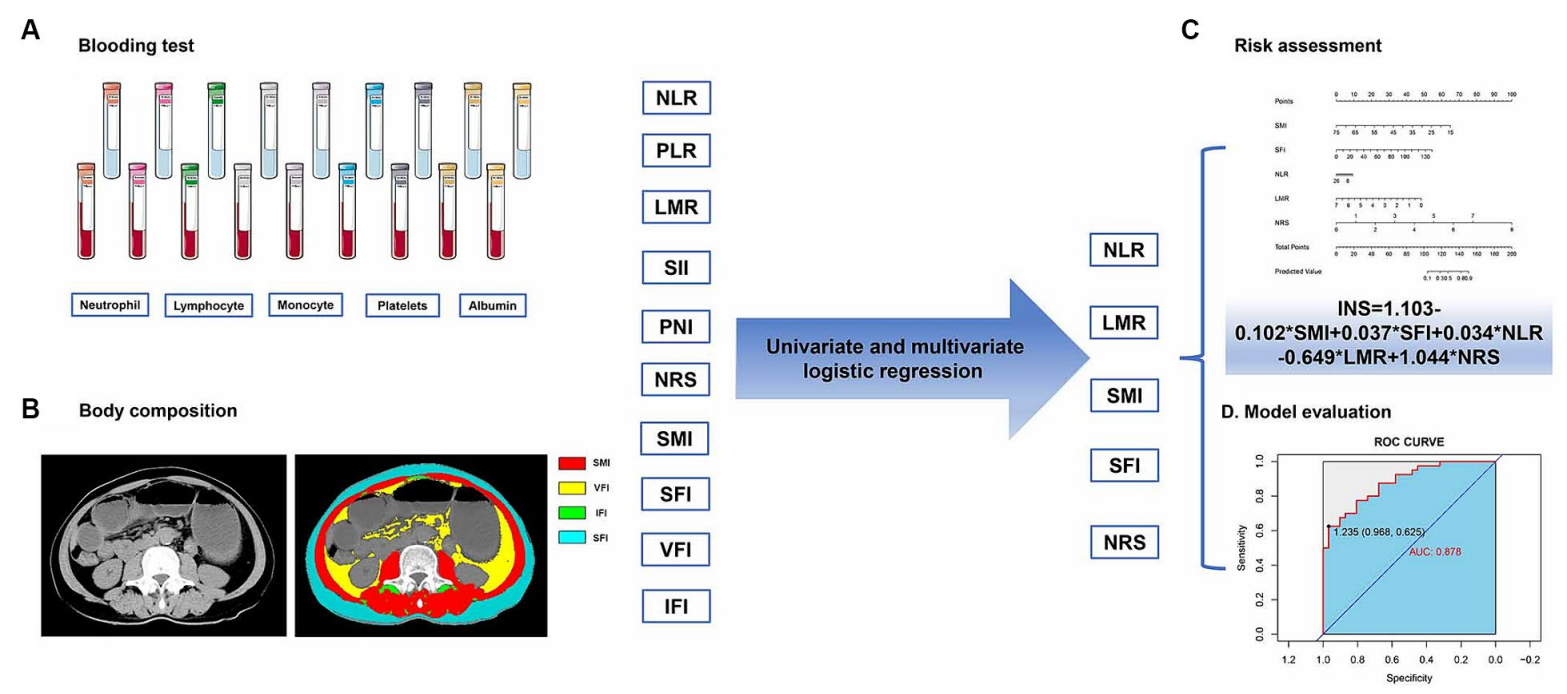
The process of analyzing inflammatory-nutritional markers, from data collection to model creation, involves: **(A)** blood test, **(B)** body composition, **(C)** risk assessment, and **(D)** model evaluation.

Using the institutional PACS (Picture Archiving and Communication System), postoperative L3 CT images were obtained for each patient. Slicer O Matic software (version 5.0)^1^ was used for assessing body composition. The CT Hounsfield units (HU) thresholds were set at −190 to −30 for intermuscular and subcutaneous adipose tissue, −150 to −50 for visceral adipose tissue, and −29 to +150 for skeletal muscle area (23). The evaluation areas were delineated by two experienced radiologists who were blinded to the clinical characteristics of the patients. The body composition indexes (cm^2^/m^2^), including skeletal muscle index (SMI), subcutaneous fat index (SFI), visceral fat index (VFI), and intermuscular adipose tissue index (IFI), were defined as the body composition area (cm^2^) by height squared (m^2^). Figure 2 presents a schematic diagram of the study workflow.

### Definitions of postoperative complications

Postoperative complications were classified according to the Clavien–Dindo classification (4). Our analysis focused on complications that occurred within 1 month after the surgical procedure. In cases where a patient experienced multiple complications either simultaneously or sequentially, the most severe complication was selected as the primary outcome for this study.

### Statistical analysis

Statistical analysis was conducted using R software version 3.6.3^2^ and SPSS version 25.0. We utilized the Kolmogorov–Smirnov test to assess the normal distribution of texture features. Intergroup categorical variables were examined using Fisher’s exact tests and Chi-square tests, while independent-sample *t*-tests were applied for continuous variables. The “rms” R package facilitated the generation of ROC curves, areas under the curves (AUCs), a nomogram, and corresponding calibration curves (24, 25). The “rmda” package was employed for decision curve analysis (DCA) (26). A *p*-value of less than 0.05 was considered statistically significant.

## Results

### Characteristics of enrolled patients

In total, 190 patients with ASBO were included in the study (96 men and 94 women; average age 62.48 ± 13.50 years). They were randomized into training (*n* = 133) and internal validation (*n* = 57) cohorts at a 7:3 ratio. An external validation cohort comprised 52 patients from another center. Basic characteristics of the three cohorts are presented in Table 1. In the training cohort, 65 patients (48.87%; 35 men and 30 women; average age 63.20 ± 13.73 years) experienced complications, compared to 26 patients (45.61%; 15 men and 11 women; average age 67.46 ± 15.85 years) in the internal validation cohort. The external validation cohort included 22 patients (42.31%; 12 men and 10 women; average age 61.23 ± 10.70 years) with complications. Factors such as preoperative infection, ASA score, NRS, intestinal strangulation, CRP, NLR, PLR, LMR, SII, PNI, SMI, and SFI showed a significant correlation with postoperative complications in the training set (*p* < 0.05).

**Table 1:** Clinical characteristics of patients in this study.

	Training set		*p* value	Internal validation set		*p* value	External validation set		*p* value
	Complications group (*n* = 65)	Non-complications group (*n* = 68)		Complications group (*n* = 26)	Non-complications group (*n* = 31)		Complications group (*n* = 22)	Non-complications group (*n* = 30)	
Age (years), mean (SD)	63.20 (13.73)	60.93 (12.48)	0.319	67.46 (15.85)	60.23 (12.46)	0.059	61.23 (10.70)	59.37 (11.61)	0.558
Gender, *n* (%)			0.300			0.790			0.575
Male	35 (53.85%)	30 (44.12%)		15 (57.69%)	16 (51.61%)		12 (54.55%)	13 (43.33%)	
Female	30 (46.15%)	38 (55.88%)		11 (42.31%)	15 (48.39%)		10 (45.45%)	17 (56.67%)	
BMI (kg/m^2^), mean (SD)	20.97 (3.48)	22.01 (3.39)	0.122	21.86 (3.69)	21.54 (3.25)	0.745	21.81 (3.83)	22.11 (2.95)	0.779
**Manifestations**									
Obstruction time (d), mean (SD)	7.32 (9.67)	9.33 (12.95)	0.313	5.77 (6.25)	6.39 (6.66)	0.724	5.68 (6.64)	5.13 (5.79)	0.752
Vomit, *n* (%)	40 (61.54%)	47 (69.12%)	0.369	14 (53.85%)	20 (64.52%)	0.432	18 (81.82%)	20 (66.67%)	0.344
Abdominal pain, *n* (%)	58 (89.23%)	66 (97.06%)	0.092	23 (88.46%)	30 (96.77%)	0.322	22 (100%)	28 (93.33%)	0.502
Abdominal distention, *n* (%)	53 (81.54%)	50 (73.53%)	0.304	20 (76.92%)	25 (80.65%)	0.755	16 (72.73%)	18 (60%)	0.390
No exhaust or defecation, *n* (%)	36 (55.38%)	33 (48.53%)	0.489	18 (69.23%)	16 (51.61%)	0.278	12 (54.55%)	11 (36.67%)	0.262
**Commodities**									
Hypertension, *n* (%)	13 (20.00%)	18 (26.47%)	0.417	7 (26.92%)	6 (19.35%)	0.541	7 (31.82%)	8 (26.67%)	0.762
Diabetes mellitus, *n* (%)	7 (10.77%)	4 (5.88%)	0.358	5 (19.23%)	3 (9.68%)	0.448	1 (4.55)	2 (6.67%)	0.999
Coronary disease, *n* (%)	4 (6.15%)	4 (5.88%)	0.999	5 (19.23%)	2 (6.45%)	0.228	3 (13.64%)	2 (6.67%)	0.639
Preoperative infection, *n* (%)	10 (15.38%)	2 (2.94%)	0.015^*^	6 (23.08%)	1 (3.23%)	0.045^*^	4 (18.18%)	2 (6.67%)	0.382
ASA score, mean (SD)	2.97 (0.59)	2.72 (0.54)	0.012^*^	3.15 (0.73)	2.74 (0.51)	0.016^*^	3.00 (0.44)	2.77 (0.43)	0.062
NRS, mean (SD)	4.08 (1.80)	2.13 (1.33)	0.001^*^	3.56 (1.67)	2.59 (1.04)	0.050^*^	4.09 (1.27)	3.17 (0.70)	0.004^*^
**Intraoperative findings**									
Enterotomy, *n* (%)	35 (53.85%)	25 (36.76%)	0.056	17 (65.38%)	15 (48.39%)	0.284	11 (50%)	8 (26.67%)	0.144
Intestinal strangulation, *n* (%)	21 (32.31%)	11 (16.18%)	0.042^*^	12 (46.15%)	6 (19.35%)	0.045*	7 (31.82%)	4 (13.33%)	0.169
HB (g/L), mean (SD)	116.83 (20.68)	116.04 (24.41)	0.841	121.15 (17.93)	124.65 (20.87)	0.505	117.91 (24.86)	125.70 (23.32)	0.253
CRP (mg/L), mean (SD)	45.50 (47.83)	22.36 (34.62)	0.003^*^	47.45 (53.73)	34.71 (57.33)	0.405	86.04 (102.24)	10.63 (18.05)	0.002^*^
NLR, mean (SD)	6.83 (5.83)	4.78 (4.27)	0.022^*^	8.41 (7.21)	5.09 (3.28)	0.037^*^	9.79 (8.64)	3.34 (4.23)	0.001^*^
PLR, mean (SD)	318.43 (226.02)	231.24 (111.59)	0.006^*^	296.09 (191.32)	224.65 (108.02)	0.099	321.80 (315.97)	181.19 (117.61)	0.058
LMR, mean (SD)	2.19 (1.43)	2.83 (1.45)	0.011^*^	2.27 (1.93)	2.75 (2.04)	0.367	2.44 (2.22)	4.30 (2.32)	0.005^*^
SII, mean (SD)	1734.83 (1692.46)	1126.24 (1060.17)	0.015^*^	2228.30 (2185.85)	1101.10 (729.05)	0.018^*^	2018.78 (1623.55)	839.09 (1494.27)	0.010^*^
PNI, mean (SD)	39.12 (6.48)	41.73 (8.17)	0.044^*^	40.31 (9.40)	40.44 (7.48)	0.953	40.16 (12.06)	45.59 (7.66)	0.053
SMI (cm^2^/m^2^), mean (SD)	33.58 (8.76)	38.86 (10.07)	0.002*	32.40 (8.64)	39.30 (9.59)	0.007^*^	32.87 (7.25)	40.58 (9.01)	0.002^*^
IFI (cm^2^/m^2^), mean (SD)	3.03 (2.37)	2.75 (2.31)	0.500	3.02 (2.88)	2.48 (2.39)	0.442	3.29 (3.25)	2.54 (2.15)	0.353
SFI (cm^2^/m^2^), mean (SD)	39.72 (25.02)	31.51 (18.59)	0.034^*^	42.44 (19.43)	28.06 (16.51)	0.004^*^	39.59 (16.98)	30.11 (14.54)	0.035^*^
VFI (cm^2^/m^2^), mean (SD)	23.16 (19.98)	23.29 (17.75)	0.968	26.12 (23.44)	24.17 (18.24)	0.726	26.44 (19.66)	18.63 (13.53)	0.095

*p < 0.05.

BMI, body mass index; ASA, American Society of Anesthesiologists; NRS, nutritional risk score; HB, hemoglobin; CRP, C-reactive protein; NLR, neutrophil-lymphocyte ratio; PLR, platelet-lymphocyte ratio; LMR, lymphocyte-monocyte ratio; SII, systemic immune inflammation index; PNI, prognostic nutritional index; SMI, skeletal muscle index; IFI, intermuscle fat index; SFI, subcutaneous fat index; VFI, visceral fat index.

### Overview of complications

The incidence of complications across different grades did not significantly differ among the three cohorts (*p* > 0.05) (Table 2). There were 37 (27.82%), 14 (24.56%), and 13 (25%) patients who experienced severe complications (Grade III or higher) in training, internal validation, and external validation cohorts, demonstrating comparable rates of severe complications.

**Table 2:** Thirty-day postoperative complications.

	Training set (*n* = 133)	Internal validation set (*n* = 57)	External validation set (*n* = 52)	*p* value
Overall postoperative complication	65 (48.87%)	26 (45.61%)	22 (42.31%)	0.711
Grade I	11 (8.27%)	5 (8.77%)	4 (7.69%)	0.979
Superficial wound infection	3	1	0	
Electrolyte imbalance	8	4	4	
Grade II	17 (12.78%)	7 (12.28%)	5 (9.62%)	0.835
Ileus (treated conservatively)	4	0	0	
Intraperitoneal hemorrhage (necessitating transfusion)	2	2	1	
Fever with antibiotics	4	2	3	
Respiratory infection	6	2	1	
Urinary infection	1	1	0	
Grade III	24 (18.05%)	6 (10.53%)	5 (9.62%)	0.214
Wound infection (necessitating reoperation)	7	1	3	
Intraperitoneal hemorrhage (necessitating reoperation)	2	2	0	
Intraperitoneal infection (necessitating reoperation)	8	1	0	
Ileus (necessitating reoperation)	3	2	1	
Intestinal fistula (necessitating reoperation)	4	0	1	
Grade IV	11 (8.27%)	7 (12.28%)	8 (15.38%)	0.340
Respiratory failure	1	1	2	
Cardiac failure	2	1	1	
Renal failure	0	1	1	
Multiple organ dysfunction syndrome	8	4	4	
Grade V	2 (1.50%)	1 (1.75%)	0	0.594
Death	2	1	0	

The definition of complications was given in the article.

### Univariate and multivariate analysis of inflammatory-nutritional markers

Table 3 indicates significant differences between the two groups in preoperative infection, ASA score, NRS, intestinal strangulation, CRP, NLR, PLR, LMR, SII, PNI, SMI, and SFI, as identified by univariate regression in the training cohort (*p* < 0.05). These variables with *p*-values less than 0.05 were subsequently included in the multivariate regression analysis. Our findings reveal that NRS (OR = 21.731, *p* = 0.002), intestinal strangulation (OR = 401.665, *p* = 0.008), NLR (OR = 4.264, *p* = 0.029), LMR (OR = 0.183, *p* = 0.034), SMI (OR = 0.708, *p* = 0.008), and SFI (OR = 1.115, *p* = 0.014) are independent predictors of postoperative complications.

**Table 3:** Univariate and multivariate analysis of patients with complications versus those without in the training set.

	Univariate analysis				Multivariate analysis			
	SE	Exp(B)	*p* value	95%CI	SE	Exp(B)	*p* value	95%CI
Preoperative infection, *n* (%)	0.796	6.000	0.024	1.261–28.547	2.360	69.815	0.072	0.684–7122.731
ASA score, mean (SD)	0.340	2.268	0.016	1.166–4.414	1.363	2.077	0.592	0.144–30.020
NRS, mean (SD)	0.219	2.278	0.001	1.484–3.497	0.995	21.731	0.002	3.092–152.746
Intestinal strangulation, *n* (%)	0.423	2.473	0.032	1.080–5.665	2.262	401.665	0.008	4.774–33797.868
CRP (mg/L), mean (SD)	0.005	1.014	0.005	1.004–1.024	0.019	1.036	0.065	0.998–1.075
NLR, mean (SD)	0.038	1.086	0.028	1.009–1.169	0.663	4.264	0.029	1.162–15.648
PLR, mean (SD)	0.001	1.004	0.008	1.001–1.006	0.005	1.004	0.388	0.994–1.015
LMR, mean (SD)	0.129	0.728	0.014	0.566–0.938	0.800	0.183	0.034	0.038–0.879
SII, mean (SD)	0.000	1.000	0.020	0.999–1.000	0.004	1.004	0.074	1.000–1.009
PNI, mean (SD)	0.025	0.952	0.049	0.906–1.000	0.112	0.834	0.105	0.670–1.039
SMI (cm^2^/m^2^), mean (SD)	0.020	0.940	0.003	0.903–0.979	0.131	0.708	0.008	0.547–0.915
SFI (cm^2^/m^2^), mean (SD)	0.008	1.018	0.037	1.001–1.035	0.044	1.115	0.014	1.022–1.217

ASA, American Society of Anesthesiologists; NRS, nutritional risk score; CRP, C-reactive protein; NLR, neutrophil-lymphocyte ratio; PLR, platelet-lymphocyte ratio; LMR, lymphocyte-monocyte ratio; SII, systemic immune inflammation index; PNI, prognostic nutritional index; SMI, skeletal muscle index; SFI, subcutaneous fat index.

### Inflammatory-nutritional model construction and verification

**Figure 3:**
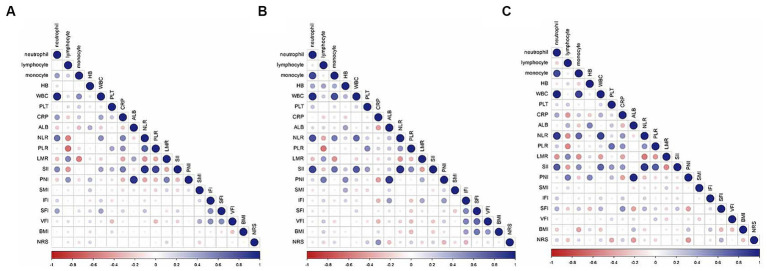
Correlation matrix of nutritional inflammatory biomarkers in the training **(A)**, internal validation **(B)**, and external validation **(C)** sets.

Figure 3 presents a correlation matrix of inflammatory-nutritional biomarkers, with correlation coefficients ranging from −1 (red) to 1 (blue) in training and validation sets. Hemoglobin (HB) was found to be correlated with SMI in both the training [Pearson Correlation Coefficient (PCC) = 0.196, *p* = 0.023] and internal validation sets (PCC = 0.348, *p* = 0.008), as shown in Supplementary Tables S1–S3. To avoid multicollinearity, the inflammatory-nutritional model was constructed using indicators with a PCC below 0.7 (27).

**Figure 4:**
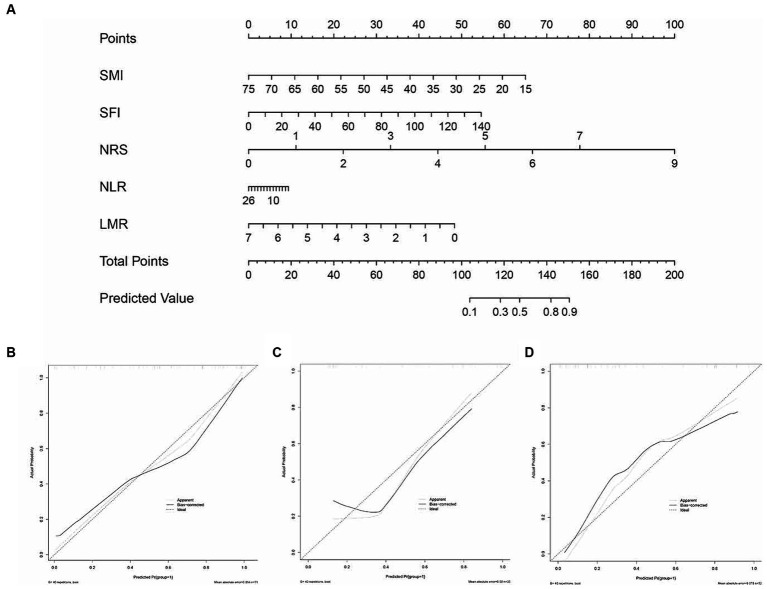
The inflammatory-nutrition nomogram and its calibration curves. **(A)** Development of the inflammatory-nutrition nomogram in the training set, incorporating SMI, SFI, NLR, PLR, and NRS. Calibration curves for the nomogram in the training **(B)**, internal validation **(C)**, and external validation **(D)** sets.

A nomogram derived from the multivariate analysis was developed, incorporating NRS, NLR, PLR, SMI, and SFI. Each patient’s total score was calculated by summing the scores of these five predictive factors, which were then used to evaluate the risk of postoperative complications. A higher total score correlated positively with an increased probability of postoperative complications (Figure 4A). The inflammatory-nutritional score (INS) was calculated as 1.103–0.102*SMI + 0.037*SFI + 0.034*NLR-0.649*LMR + 1.044*NRS. Calibration curves demonstrated good agreement in three cohorts (Figures 4B–D). Validation was conducted using the bootstrap method, and model performance was assessed over 1,000 iterations.

### Evaluating predictive performance of the three models

**Figure 5:**
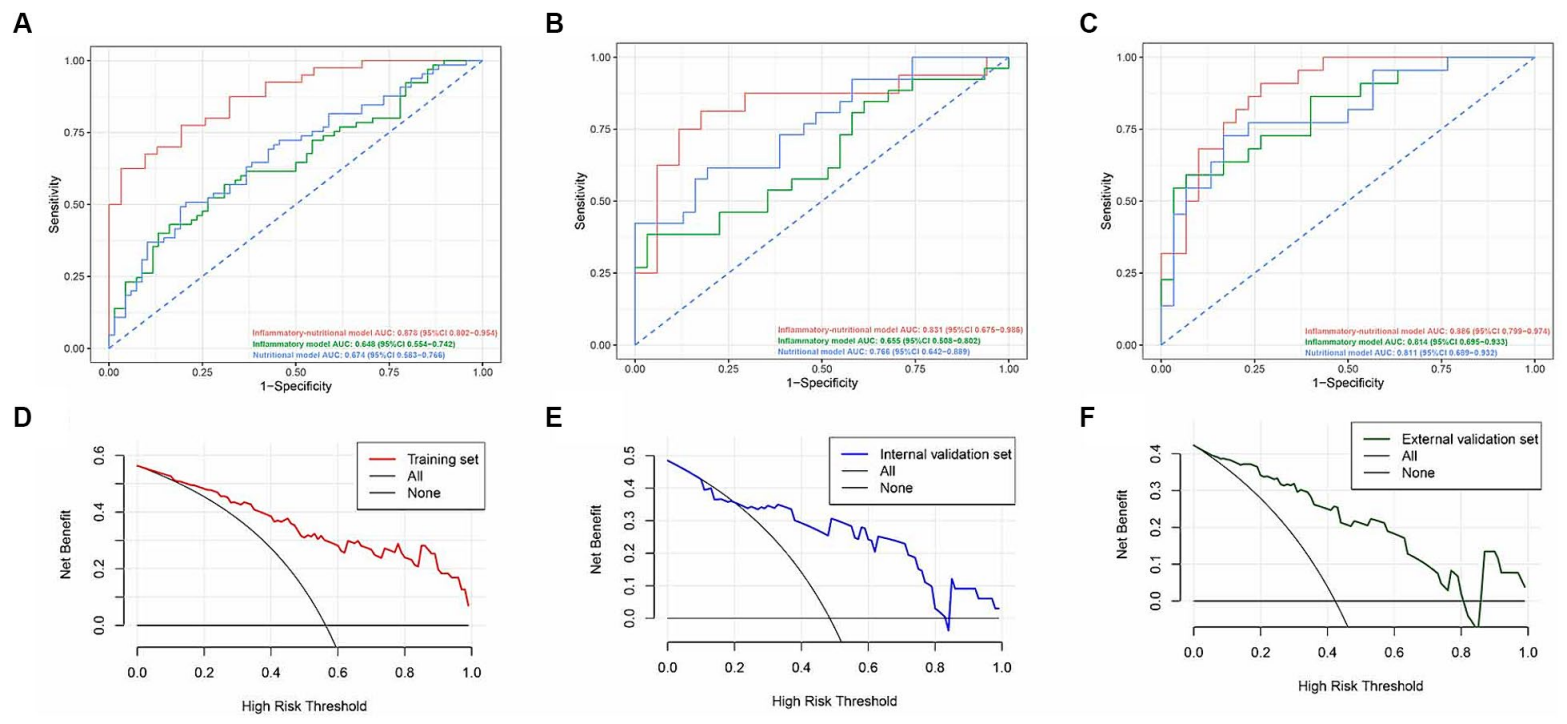
Receiver operating characteristic curves of the inflammatory-nutrition model, the inflammatory model, and the nutritional model in the training **(A)**, internal validation **(B)**, and external validation **(C)** sets. Decision curve analysis for the inflammatory-nutrition model in the training **(D)**, internal validation **(E)**, and external validation **(F)** sets.

Based on inflammatory-nutritional markers, we established an inflammatory model (NLR, PLR, LMR, SII, PNI) and nutritional model (SMI, IFI, SFI, VFI). ROC analysis revealed that the nomogram achieved AUCs of 0.878 (95% CI, 0.802–0.954) in the training set, 0.831 (95% CI, 0.675–0.986) in the internal validation set, and 0.886 (95% CI, 0.799–0.974) in the external validation set. These results surpassed those of the inflammatory model (0.648, 95% CI 0.554–0.742) and the nutritional model (0.674, 95% CI 0.583–0.766) in the training set, 0.655 (95% CI 0.508–0.802) and 0.766 (95% CI 0.642–0.889) in the internal validation set, and 0.814 (95% CI 0.695–0.933) and 0.811 (95% CI 0.689–0.932) respectively (Figures 5A–C) in the external validation set. Decision curve analysis (DCA) indicated that our nomogram achieved greater net benefits at optimal threshold probabilities in predicting complications in ASBO cases (Figures 5D–F). Table 4 details the predictive performance of the inflammatory model, nutritional model, and nomogram in three cohorts.

**Table 4:** Predictive performance of the three models in the training and validation sets.

	Training set (*n* = 133)						Internal validation set (*n* = 57)						External validation set (*n* = 52)					
	AUC (95%CI)	ACC	SEN	SPE	PPV	NPV	AUC (95%CI)	ACC	SEN	SPE	PPV	NPV	AUC (95%CI)	ACC	SEN	SPE	PPV	NPV
Inflammatory model	0.648 (95%CI 0.554–0.742)	0.639	0.431	0.838	0.718	0.606	0.655 (95%CI 0.508–0.802)	0.702	0.385	0.968	0.909	0.652	0.814 (95%CI 0.695–0.933)	0.788	0.591	0.933	0.867	0.757
Nutritional model	0.674 (95%CI 0.583–0.766)	0.654	0.508	0.794	0.702	0.628	0.766 (95%CI 0.642–0.889)	0.737	0.423	0.999	0.999	0.674	0.811 (95%CI 0.689–0.932)	0.788	0.727	0.833	0.762	0.806
Inflammatory-nutritional model	0.878 (95%CI 0.802–0.954)	0.755	0.625	0.968	0.962	0.667	0.831 (95%CI 0.675–0.986)	0.818	0.812	0.824	0.812	0.824	0.886 (95%CI 0.799–0.974)	0.808	0.909	0.733	0.714	0.917

AUC, area under the curve; CI, confidence interval; ACC, accuracy; SEN, sensitivity; SPE, specificity; PPV, positive predictive value; NPV, negative predictive value.

## Discussion

The choice between conservative management and surgical intervention for ASBO patients continues to be debated. Previous research indicated that the method of treatment correlates with postoperative complications in ASBO patients (28, 29). In our study, 113 of the 242 ASBO patients (46.69%) encountered complications (30). This significant rate of postoperative complications adversely affects ASBO patient outcomes, underscoring the necessity for a predictive model to foresee these complications and assist in clinical decision-making (31). Our current research, involving 242 ASBO patients, validated the efficacy of a nomogram that incorporates CT-based body composition and inflammatory-nutritional markers. This model, easy to compute, holds broad applicability in clinical practice.

Increasing evidence suggests that poor nutritional status is a prognostic risk factor for various gastrointestinal disorders, encompassing both malignancies and benign conditions (14, 32, 33). In ASBO cases, impaired intestinal function curtails the efficacy of standard enteral interventions in swiftly rectifying malnutrition, elevating the risk of severe malnutrition and impeding potential enhancements in nutritional status due to acute gastrointestinal failure (34). Consequently, clinicians are in pursuit of reliable indicators to accurately evaluate the nutritional status of ASBO patients (35). Although traditional nutritional assessment tools like body weight and BMI offer a general insight into an individual’s nutritional status, they do not provide specific details on body composition, such as muscle mass or regional fat distribution. Recent research has increasingly acknowledged the pivotal role of body composition in determining a patient’s nutritional state and postoperative outcomes (36, 37). Our study also showed that the SMIs of ASBO patients with complications were significantly lower than those of patients without complications (*p* < 0.05), and multivariable analysis confirmed this protective factor (OR = 0.708). This association indicated the importance of maintaining skeletal muscle mass quality for postoperative recovery of ASBO patients (38). Patients at a heightened risk of sarcopenia may undergo a persistent inflammatory response that disrupts normal nitrogen metabolism, increasing the likelihood of postoperative complications. This is consistent with prior findings that surgical patients with reduced skeletal muscle mass have poorer prognoses, encounter more postoperative complications, require more intensive care, and exhibit higher mortality rates (39, 40). Furthermore, our observations indicate that a high SFI correlates with postoperative complications in ASBO patients, adding to the discourse on the “obesity paradox” and supporting the notion that sarcopenic obesity is as indicative of surgical outcomes as sarcopenia alone, as several studies have previously reported (41, 42).

ASBO is often associated with acute inflammatory process, a key marker of disease progression. Mueller et al. discovered that as obstruction advances and intestinal barrier dysfunction ensues, luminal flora can penetrate the mucosal layer, triggering host immune responses (43). This uncontrolled inflammatory response is characterized by immune cells infiltration and the release of inflammatory mediators. Numerous systemic inflammatory response indicators, derived from serum biomarkers, have been developed to assess the extent of this response. In our study, systemic inflammatory response indicators, including CRP, PLR, NLR, LMR, PNI and SII, were examined. We found that patients with higher NLR and lower LMR values were more likely to experience postoperative complications, corroborating previous findings that NLR and LMR are crucial indicators for predicting disease severity and prognosis in cancer patients (12, 44, 45). NLR and LMR calculations involve neutrophils, lymphocytes, and monocytes. Changes in NLR and LMR values generally reflect disturbances in these immune cell types and their prognostic significance, linked to the effects of such cells. Neutrophil and monocyte activation, a response to infection signals, is a fundamental component of the innate immune response phase and is implicated in the pathogenesis of various diseases (46). A decrease in lymphocyte count, indicating impaired immune function, hampers the body’s ability to combat persistent infections (11, 47, 48). In recent years, the interplay between inflammation and nutrition has gained significant attention. The volume of research examining the impact of nutrition on the immune system is continuously expanding (49, 50). On correlation analysis, we also found HB and SMI were positively correlated (Supplementary Table S1) which implied the underlying mechanistic association between anemia and sarcopenia in ASBO settings. Consistent with the findings of Hirani’s study, lower HB levels might contribute to a decrease in skeletal muscle volume, through biological pathways generally involving decreased oxygenation of skeletal muscle tissues (51). Anemia caused by ASBO may impair oxygen delivery and expenditure within muscle tissues, creating hypoxia within the local microenvironment that undermines the function of skeletal muscle cells, ultimately leading to skeletal muscle loss and sarcopenia.

The findings from our multivariate and correlation analyses have paved the way for the development of a practical predictive model for postoperative complications in ASBO patients. This model aims to identify those at heightened risk for such complications. While most previous studies have focused on the predictive power of single inflammatory–nutritional scores or CT composition indices on prognosis (45, 52), these singular measures often fail to provide a comprehensive and accurate representation of a patient’s entire inflammatory-nutritional status, thereby limiting their practical accuracy (21). Recently, there has been a trend toward developing prognostic scores based on multiple inflammatory-nutrition indices. For instance, Wang et al. created a prognostic score incorporating LMR, NLR, and PLR to predict outcomes for gastrectomy patients’ post-chemotherapy, with their nomogram demonstrating superior predictive performance (C-index 0.707) compared to single-index models (53). Our previous research utilized multiple inflammatory-nutritional scores to construct a model for predicting postoperative quality of life in gastric cancer patients (22). In this study, we initially attempted to create an inflammation-based model (Inflam-model) using inflammatory scores (Inflam-scores) and a radiography-based model (Radio-model) using body composition parameters. However, both models exhibited suboptimal performance. Consequently, we explored whether combining various inflammatory factors with nutrition-related indicators could enhance the predictive accuracy for ASBO patients. We selected significant inflammatory-nutritional and radiographic indicators from the multivariable analysis to construct a combined predictive model. Our risk predictive model, integrating two Inflam-scores, two Radio-scores, and NRS, showed improved performance in both cohorts. These Radio-scores and Inflam-scores, derived from routine clinical practice, make this multiparametric model practical, particularly in preoperative settings. Identifying patients with a high inflammatory state and low nutritional status preoperatively is crucial in clinical practice. Accordingly, prognosis may be improved through prompt and effective therapeutic interventions.

This study has several limitations. Firstly, the sample size was comparatively small, indicating the necessity for subsequent multicenter studies with expanded sample sizes. Secondly, the expansion of the intestine lumen within the abdominal cavity may affect the accuracy of visceral adipose tissue detection on CT images. Relying solely on measurements of visceral adipose tissue in single CT slides may not sufficiently predict postoperative outcomes. Dynamic and comprehensive assessments of whole-body composition warrant further study. Thirdly, although the correlation between the inflammatory and nutritional factors identified in ultimate model was investigated initially, the causal relationship of these factors is unknown, which might have impact on clinical management. Some promising new statistical methods could assist in quantifying robustness of causal inferences in future research (54).

## Conclusion

In summary, we have developed and validated a nomogram that incorporates CT body composition data and inflammatory–nutritional scores to predict postoperative complications in patients with ASBO. Given its usability and the positive results achieved in our initial cohort, this model demonstrates potential as an effective tool for guiding nutritional treatment and decision-making in ASBO cases in future clinical settings.

## Data availability statement

The original contributions presented in the study are included in the article/Supplementary material, further inquiries can be directed to the corresponding authors.

## Author contributions

ZW: Data curation, Writing – original draft. BS: Formal analysis, Methodology, Writing – original draft. YY: Formal analysis, Writing – original draft. JL: Validation, Writing – original draft. DL: Formal analysis, Writing – review & editing. YL: Writing – review & editing. RL: Funding acquisition, Writing – review & editing.

## Ethics statement

The studies involving humans were approved by The Affiliated Hospital of Qingdao University. The studies were conducted in accordance with the local legislation and institutional requirements. Written informed consent for participation was not required from the participants or the participants’ legal guardians/next of kin in accordance with the national legislation and institutional requirements.

## Footnotes

^1^http://www.tomovision.com

^2^https://www.r-project.org

## Glossary

ASBO: Adhesive small bowel obstruction
NLR: Neutrophil-lymphocyte ratio
PLR: Platelet–lymphocyte ratio
LMR: Lymphocyte-monocyte ratio
SII: Systemic immune-inflammation index
PNI: Prognostic nutritional index
CRP: C-reactive protein
BMI: Body mass index
CT: Computed tomography
ASA: American society of anesthesiologists
NRS: Nutritional risk score
SMI: Skeletal muscle index
SFI: Subcutaneous fat index
IFI: Intermuscular fat index
VFI: Visceral fat index
INS: Inflammatory-nutritional score
ROC: Receiver-operating characteristic
DCA: Decision curve analysis
OR: Odds ratio
