# Supplementary material for: A novel nomogram integrating body composition and inflammatory-nutritional markers for predicting postoperative complications in patients with adhesive small bowel obstruction
Source: Front Nutr. 2024 Apr 19;11:1345570. doi: 10.3389/fnut.2024.1345570 (PMC11066162; doi:10.3389/fnut.2024.1345570)
Supplement: Supplementary file 1 [file Table_1.docx]

**Table S1 Inflammatory-nutrition markers correlation matrix in the training set**

|  | neutrophil | lymphocyte | monocyte | HB | WBC | PLT | CRP | ALB | NLR | PLR | LMR | SII | PNI | SMI | IFI | SFI | VFI | BMI | NRS |
| --- | --- | --- | --- | --- | --- | --- | --- | --- | --- | --- | --- | --- | --- | --- | --- | --- | --- | --- | --- |
| neutrophil | 1 | 0.030 | .480** | .240** | .976** | .206* | .312** | 0.002 | .643** | 0.051 | -.302** | .659** | -0.001 | -0.040 | 0.010 | 0.142 | -0.008 | -0.064 | 0.013 |
| lymphocyte | 0.030 | 1 | .181* | 0.080 | .196* | .231** | -.250** | 0.129 | -.518** | -.644** | .552** | -.389** | .474** | 0.106 | 0.015 | -0.021 | 0.117 | 0.029 | 0.117 |
| monocyte | .480** | .181* | 1 | 0.108 | .549** | .290** | 0.129 | -.208* | 0.130 | -0.097 | -.547** | .202* | -0.086 | 0.048 | -0.015 | 0.004 | 0.041 | -0.042 | -0.046 |
| HB | .240** | 0.080 | 0.108 | 1 | .235** | 0.044 | -.219* | .511** | 0.077 | -0.031 | 0.050 | 0.137 | .402** | .196* | -0.035 | 0.108 | 0.116 | -0.095 | 0.115 |
| WBC | .976** | .196* | .549** | .235** | 1 | .262** | .265** | -0.004 | .532** | -0.056 | -.237** | .575** | 0.058 | -0.029 | 0.013 | 0.119 | 0.000 | -0.043 | 0.059 |
| PLT | .206* | .231** | .290** | 0.044 | .262** | 1 | 0.147 | 0.060 | 0.003 | .306** | -0.039 | .338** | 0.160 | -0.099 | -0.011 | -0.045 | -0.113 | 0.085 | 0.018 |
| CRP | .312** | -.250** | 0.129 | -.219* | .265** | 0.147 | 1 | -.294** | .490** | .433** | -.324** | .488** | -.297** | -0.147 | 0.050 | 0.095 | -0.063 | -0.044 | 0.008 |
| ALB | 0.002 | 0.129 | -.208* | .511** | -0.004 | 0.060 | -.294** | 1 | -0.111 | -0.072 | .310** | -0.023 | .913** | 0.062 | -0.082 | -0.062 | -0.060 | -0.083 | -0.102 |
| NLR | .643** | -.518** | 0.130 | 0.077 | .532** | 0.003 | .490** | -0.111 | 1 | .607** | -.496** | .892** | -.280** | -0.116 | -0.016 | 0.089 | -0.129 | -0.055 | 0.028 |
| PLR | 0.051 | -.644** | -0.097 | -0.031 | -0.056 | .306** | .433** | -0.072 | .607** | 1 | -.440** | .717** | -.276** | -.204* | -0.065 | -0.002 | -.225** | -0.072 | 0.051 |
| LMR | -.302** | .552** | -.547** | 0.050 | -.237** | -0.039 | -.324** | .310** | -.496** | -.440** | 1 | -.436** | .451** | 0.145 | -0.044 | -0.063 | 0.048 | 0.008 | 0.100 |
| SII | .659** | -.389** | .202* | 0.137 | .575** | .338** | .488** | -0.023 | .892** | .717** | -.436** | 1 | -0.155 | -0.121 | -0.041 | 0.066 | -0.159 | -0.054 | 0.073 |
| PNI | -0.001 | .474** | -0.086 | .402** | 0.058 | 0.160 | -.297** | .913** | -.280** | -.276** | .451** | -0.155 | 1 | 0.099 | -0.046 | -0.017 | 0.029 | -0.081 | -0.042 |
| SMI | -0.040 | 0.106 | 0.048 | .196* | -0.029 | -0.099 | -0.147 | 0.062 | -0.116 | -.204* | 0.145 | -0.121 | 0.099 | 1 | 0.113 | -0.135 | .288** | -0.007 | -0.172 |
| IFI | 0.010 | 0.015 | -0.015 | -0.035 | 0.013 | -0.011 | 0.050 | -0.082 | -0.016 | -0.065 | -0.044 | -0.041 | -0.046 | 0.113 | 1 | .457** | .556** | -0.002 | 0.017 |
| SFI | 0.142 | -0.021 | 0.004 | 0.108 | 0.119 | -0.045 | 0.095 | -0.062 | 0.089 | -0.002 | -0.063 | 0.066 | -0.017 | -0.135 | .457** | 1 | .503** | -0.055 | -0.022 |
| VFI | -0.008 | 0.117 | 0.041 | 0.116 | 0.000 | -0.113 | -0.063 | -0.060 | -0.129 | -.225** | 0.048 | -0.159 | 0.029 | .288** | .556** | .503** | 1 | -0.125 | -0.063 |
| BMI | -0.064 | 0.029 | -0.042 | -0.095 | -0.043 | 0.085 | -0.044 | -0.083 | -0.055 | -0.072 | 0.008 | -0.054 | -0.081 | -0.007 | -0.002 | -0.055 | -0.125 | 1 | -0.088 |
| NRS | 0.013 | 0.117 | -0.046 | 0.115 | 0.059 | 0.018 | 0.008 | -0.102 | 0.028 | 0.051 | 0.100 | 0.073 | -0.042 | -0.172 | 0.017 | -0.022 | -0.063 | -0.088 | 1 |
| *P<0.05 **P<0.01 |  |  |  |  |  |  |  |  |  |  |  |  |  |  |  |  |  |  |  |

**Table S2 Inflammatory-nutrition markers correlation matrix in the internal validation set**

|  | neutrophil | lymphocyte | monocyte | HB | WBC | PLT P | CRP | ALB | NLR | PLR | LMR | SII | PNI | SMI | IFI | SFI | VFI | BMI | NRS |
| --- | --- | --- | --- | --- | --- | --- | --- | --- | --- | --- | --- | --- | --- | --- | --- | --- | --- | --- | --- |
| neutrophil | 1 | 0.019 | .776** | .345** | .955** | 0.206 | 0.062 | -0.022 | .804** | 0.077 | -.300* | .843** | -0.011 | -0.169 | 0.174 | 0.165 | -0.029 | -0.046 | 0.093 |
| lymphocyte | 0.019 | 1 | 0.107 | .303* | 0.151 | -0.045 | -.313* | 0.238 | -.424** | -.598** | .533** | -.324* | .578** | 0.210 | -0.029 | 0.013 | 0.196 | 0.109 | -0.250 |
| monocyte | .776** | 0.107 | 1 | 0.200 | .782** | 0.179 | 0.146 | -.309* | .502** | -0.068 | -.450** | .597** | -0.219 | 0.009 | -0.081 | 0.018 | -0.101 | -0.142 | 0.125 |
| HB | .345** | .303* | 0.200 | 1 | .317* | 0.042 | -.358** | .451** | 0.185 | -0.101 | 0.122 | 0.224 | .494** | .348** | 0.119 | 0.044 | 0.130 | 0.197 | 0.007 |
| WBC | .955** | 0.151 | .782** | .317* | 1 | 0.165 | 0.019 | -0.048 | .694** | -0.040 | -0.227 | .755** | 0.016 | -0.156 | 0.153 | 0.209 | 0.088 | -0.055 | 0.051 |
| PLT P | 0.206 | -0.045 | 0.179 | 0.042 | 0.165 | 1 | 0.112 | -0.016 | 0.131 | .569** | -0.130 | .456** | -0.030 | -0.124 | -0.089 | 0.073 | -0.123 | 0.028 | 0.244 |
| CRP | 0.062 | -.313* | 0.146 | -.358** | 0.019 | 0.112 | 1 | -.563** | 0.205 | .294* | -.328* | 0.194 | -.591** | -0.113 | -0.048 | -0.039 | -0.239 | 0.089 | .402* |
| ALB | -0.022 | 0.238 | -.309* | .451** | -0.048 | -0.016 | -.563** | 1 | -0.018 | 0.020 | .402** | -0.032 | .930** | 0.136 | 0.213 | 0.012 | 0.115 | 0.142 | -0.210 |
| NLR | .804** | -.424** | .502** | 0.185 | .694** | 0.131 | 0.205 | -0.018 | 1 | .457** | -.442** | .893** | -0.175 | -0.182 | 0.257 | 0.213 | -0.097 | -0.128 | 0.141 |
| PLR | 0.077 | -.598** | -0.068 | -0.101 | -0.040 | .569** | .294* | 0.020 | .457** | 1 | -.340** | .567** | -0.209 | -0.092 | -0.091 | -0.063 | -0.253 | -0.219 | 0.240 |
| LMR | -.300* | .533** | -.450** | 0.122 | -0.227 | -0.130 | -.328* | .402** | -.442** | -.340** | 1 | -.425** | .539** | -0.013 | 0.113 | 0.013 | 0.042 | 0.010 | -0.158 |
| SII | .843** | -.324* | .597** | 0.224 | .755** | .456** | 0.194 | -0.032 | .893** | .567** | -.425** | 1 | -0.149 | -0.222 | 0.076 | 0.152 | -0.171 | -0.155 | 0.199 |
| PNI | -0.011 | .578** | -0.219 | .494** | 0.016 | -0.030 | -.591** | .930** | -0.175 | -0.209 | .539** | -0.149 | 1 | 0.193 | 0.168 | 0.015 | 0.171 | 0.159 | -0.286 |
| SMI | -0.169 | 0.210 | 0.009 | .348** | -0.156 | -0.124 | -0.113 | 0.136 | -0.182 | -0.092 | -0.013 | -0.222 | 0.193 | 1 | -0.109 | -0.206 | 0.118 | 0.054 | -0.221 |
| IFI | 0.174 | -0.029 | -0.081 | 0.119 | 0.153 | -0.089 | -0.048 | 0.213 | 0.257 | -0.091 | 0.113 | 0.076 | 0.168 | -0.109 | 1 | .449** | .412** | .431** | -0.300 |
| SFI | 0.165 | 0.013 | 0.018 | 0.044 | 0.209 | 0.073 | -0.039 | 0.012 | 0.213 | -0.063 | 0.013 | 0.152 | 0.015 | -0.206 | .449** | 1 | .603** | .429** | 0.051 |
| VFI | -0.029 | 0.196 | -0.101 | 0.130 | 0.088 | -0.123 | -0.239 | 0.115 | -0.097 | -0.253 | 0.042 | -0.171 | 0.171 | 0.118 | .412** | .603** | 1 | .495** | -0.211 |
| BMI | -0.046 | 0.109 | -0.142 | 0.197 | -0.055 | 0.028 | 0.089 | 0.142 | -0.128 | -0.219 | 0.010 | -0.155 | 0.159 | 0.054 | .431** | .429** | .495** | 1 | 0.090 |
| NRS | 0.093 | -0.250 | 0.125 | 0.007 | 0.051 | 0.244 | .402* | -0.210 | 0.141 | 0.240 | -0.158 | 0.199 | -0.286 | -0.221 | -0.300 | 0.051 | -0.211 | 0.090 | 1 |
| *P<0.05 **P<0.01. |  |  |  |  |  |  |  |  |  |  |  |  |  |  |  |  |  |  |  |

|  | neutrophil | lymphocyte | monocyte | HB | WBC | PLT P | CRP | ALB | NLR | PLR | LMR | SII | PNI | SMI | IFI | SFI | VFI | BMI | NRS |
| --- | --- | --- | --- | --- | --- | --- | --- | --- | --- | --- | --- | --- | --- | --- | --- | --- | --- | --- | --- |
| neutrophil | 1 | 0.019 | .776** | .345** | .955** | 0.206 | 0.062 | -0.022 | .804** | 0.077 | -.300* | .843** | -0.011 | -0.169 | 0.174 | 0.165 | -0.029 | -0.046 | 0.093 |
| lymphocyte | 0.019 | 1 | 0.107 | .303* | 0.151 | -0.045 | -.313* | 0.238 | -.424** | -.598** | .533** | -.324* | .578** | 0.210 | -0.029 | 0.013 | 0.196 | 0.109 | -0.250 |
| monocyte | .776** | 0.107 | 1 | 0.200 | .782** | 0.179 | 0.146 | -.309* | .502** | -0.068 | -.450** | .597** | -0.219 | 0.009 | -0.081 | 0.018 | -0.101 | -0.142 | 0.125 |
| HB | .345** | .303* | 0.200 | 1 | .317* | 0.042 | -.358** | .451** | 0.185 | -0.101 | 0.122 | 0.224 | .494** | .348** | 0.119 | 0.044 | 0.130 | 0.197 | 0.007 |
| WBC | .955** | 0.151 | .782** | .317* | 1 | 0.165 | 0.019 | -0.048 | .694** | -0.040 | -0.227 | .755** | 0.016 | -0.156 | 0.153 | 0.209 | 0.088 | -0.055 | 0.051 |
| PLT P | 0.206 | -0.045 | 0.179 | 0.042 | 0.165 | 1 | 0.112 | -0.016 | 0.131 | .569** | -0.130 | .456** | -0.030 | -0.124 | -0.089 | 0.073 | -0.123 | 0.028 | 0.244 |
| CRP | 0.062 | -.313* | 0.146 | -.358** | 0.019 | 0.112 | 1 | -.563** | 0.205 | .294* | -.328* | 0.194 | -.591** | -0.113 | -0.048 | -0.039 | -0.239 | 0.089 | .402* |
| ALB | -0.022 | 0.238 | -.309* | .451** | -0.048 | -0.016 | -.563** | 1 | -0.018 | 0.020 | .402** | -0.032 | .930** | 0.136 | 0.213 | 0.012 | 0.115 | 0.142 | -0.210 |
| NLR | .804** | -.424** | .502** | 0.185 | .694** | 0.131 | 0.205 | -0.018 | 1 | .457** | -.442** | .893** | -0.175 | -0.182 | 0.257 | 0.213 | -0.097 | -0.128 | 0.141 |
| PLR | 0.077 | -.598** | -0.068 | -0.101 | -0.040 | .569** | .294* | 0.020 | .457** | 1 | -.340** | .567** | -0.209 | -0.092 | -0.091 | -0.063 | -0.253 | -0.219 | 0.240 |
| LMR | -.300* | .533** | -.450** | 0.122 | -0.227 | -0.130 | -.328* | .402** | -.442** | -.340** | 1 | -.425** | .539** | -0.013 | 0.113 | 0.013 | 0.042 | 0.010 | -0.158 |
| SII | .843** | -.324* | .597** | 0.224 | .755** | .456** | 0.194 | -0.032 | .893** | .567** | -.425** | 1 | -0.149 | -0.222 | 0.076 | 0.152 | -0.171 | -0.155 | 0.199 |
| PNI | -0.011 | .578** | -0.219 | .494** | 0.016 | -0.030 | -.591** | .930** | -0.175 | -0.209 | .539** | -0.149 | 1 | 0.193 | 0.168 | 0.015 | 0.171 | 0.159 | -0.286 |
| SMI | -0.169 | 0.210 | 0.009 | .348** | -0.156 | -0.124 | -0.113 | 0.136 | -0.182 | -0.092 | -0.013 | -0.222 | 0.193 | 1 | -0.109 | -0.206 | 0.118 | 0.054 | -0.221 |
| IFI | 0.174 | -0.029 | -0.081 | 0.119 | 0.153 | -0.089 | -0.048 | 0.213 | 0.257 | -0.091 | 0.113 | 0.076 | 0.168 | -0.109 | 1 | .449** | .412** | .431** | -0.300 |
| SFI | 0.165 | 0.013 | 0.018 | 0.044 | 0.209 | 0.073 | -0.039 | 0.012 | 0.213 | -0.063 | 0.013 | 0.152 | 0.015 | -0.206 | .449** | 1 | .603** | .429** | 0.051 |
| VFI | -0.029 | 0.196 | -0.101 | 0.130 | 0.088 | -0.123 | -0.239 | 0.115 | -0.097 | -0.253 | 0.042 | -0.171 | 0.171 | 0.118 | .412** | .603** | 1 | .495** | -0.211 |
| BMI | -0.046 | 0.109 | -0.142 | 0.197 | -0.055 | 0.028 | 0.089 | 0.142 | -0.128 | -0.219 | 0.010 | -0.155 | 0.159 | 0.054 | .431** | .429** | .495** | 1 | 0.090 |
| NRS | 0.093 | -0.250 | 0.125 | 0.007 | 0.051 | 0.244 | .402* | -0.210 | 0.141 | 0.240 | -0.158 | 0.199 | -0.286 | -0.221 | -0.300 | 0.051 | -0.211 | 0.090 | 1 |
| *P<0.05 **P<0.01. |  |  |  |  |  |  |  |  |  |  |  |  |  |  |  |  |  |  |  |

**Table S3 Inflammatory-nutrition markers correlation matrix in the external validation set**
